# Supplementary material for: Deficiency of Caspase-1 Attenuates HIV-1-Associated Atherogenesis in Mice
Source: Int J Mol Sci. 2023 Aug 17;24(16):12871. doi: 10.3390/ijms241612871 (PMC10454548; doi:10.3390/ijms241612871)
Supplement: Supplementary file 1 [file ijms-24-12871-s001.zip › ijms-2518150-supplementary.pdf]

Supplementary Material

Deficiency of Caspase-1 Attenuates HIV-1-Associated Atherogenesis in Mice

Mohammad Afaque Alam [1,2,†], Maurizio Caocci [3,†], Mi Ren [1,2], Zheng Chen [1,2], Fengming Liu [1,2], Mst Shamima Khatun [4], Jay K. Kolls [4,5], Xuebin Qin [1,2,*] and Tricia H. Burdo [3,*]

[1] Department of Comparative Pathology, Tulane National Primate Research Center, Tulane University School of Medicine, Tulane University, 18703 Three Rivers Road, Covington, LA 70433, USA;
malam1@tulane.edu (M.A.A.); mrem@tulane.edu (M.R.); zchen29@tulane.edu (Z.C.); liufengming329@gmail.com (F.L.)

[2] Department of Microbiology and Immunology, School of Medicine, Tulane University, New Orleans, LA 70112, USA

[3] Department of Microbiology, Immunology and Inflammation, Center for NeuroVirology and Gene Editing, Lewis Katz School of Medicine, Temple University, Philadelphia, PA 19140, USA; tuh20265@temple.edu

[4] Departments of Pediatrics & Medicine, Center for Translational Research in Infection and Inflammation, Tulane University School of Medicine, New Orleans, LA 70112, USA; mkhatun@tulane.edu (M.S.K.); jkolls1@tulane.edu (J.K.K.)

[5] Department of Medicine, Section of Pulmonary Diseases, Critical Care and Environmental Medicine, Tulane University School of Medicine, New Orleans, LA 70112, USA

* Correspondence: xqin2@tulane.edu (X.Q.); burdot@temple.edu (T.H.B.); Tel.: +1-215-707-1618 (T.H.B.)

† These authors contributed equally to this work.

Table S1. Antibodies and reagents.

Antibodies and reagents	Company	Cat. No.
Anti-Mouse CD11b PE-Cyanine7, M1/70	eBioscience	25-0112-81
APC anti-mouse Ly-6G Antibody	Biolegend	127613
Anti-Mouse Ly-6C eFluor® 450	eBioscience	48-5932-80
FAM-FLICA	Immunochemistry Technology	97
Propidium Iodide	Immunochemistry Technology	638
CD68 antibody	Invitrogen	14-0681-82
CD3 Antibody (PC3/188A) FITC	Santa Cruz Biotechnology	sc-20047 FITC
CD163 antibody [EPR19518]	abcam	ab182422
M-CSF	Millipore Sigma	M6518-10UG
OxLDL	Kalen biomedical, LLC	770202-4
DAPI Solution	Thermo Scientific	62248
Caspase-1 (M-19)	Santa Cruz Biotechnology	sc-1218R
Goat anti-rat-IgG	Invitrogen	A-11006
Goat anti-rabbit-HRP	DAKO	P0448

Abbreviations: FAM-FLICA: Fluorescein amidites-labeled inhibitors of caspase; M-CSF: Macrophage colony-stimulating factor; oxLDL: oxidized low-density-lipoproteins; HRP: horseradish peroxidase.

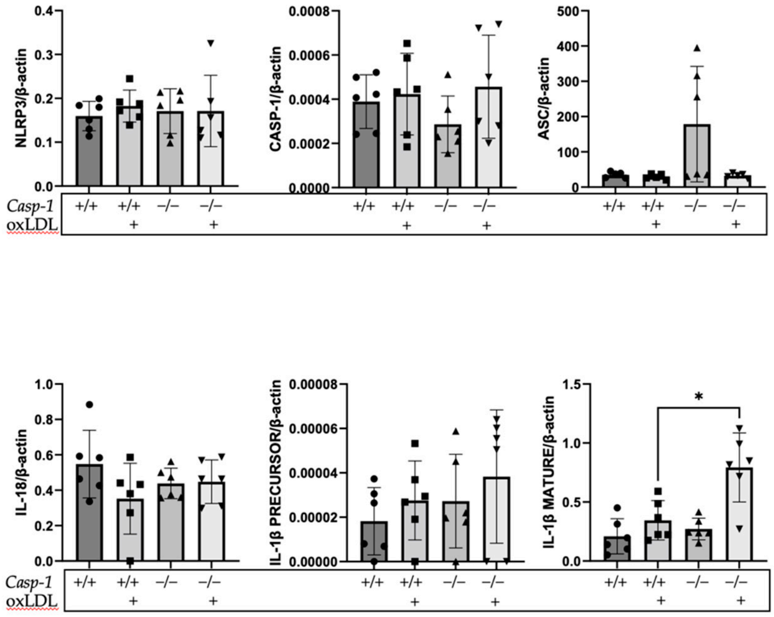

Figure S1. NRLP3 inflammasome pathway activation in MDMs from spleen of *Tg26*$^{+/-}$/*ApoE*$^{-/-}$/*Casp-1*$^{+/+}$ and *Tg26*$^{+/-}$/*ApoE*$^{-/-}$/*Casp-1*$^{-/-}$ mice after 12 weeks of an atherogenic diet. Monocyte-derived macrophages (MDM) cells isolated from spleens of caspase-1 sufficient and caspase-1 deficient mice, were cultured for 8 days and incubated with/without oxLDL (100 µg/mL) for 24 hours. mRNA levels were analyzed by RT-qPCR, normalized to β-actin, and compared between groups. Levels between all groups were analyzed using one-way ANOVA, and post-hoc t-tests were used. *p<0.05

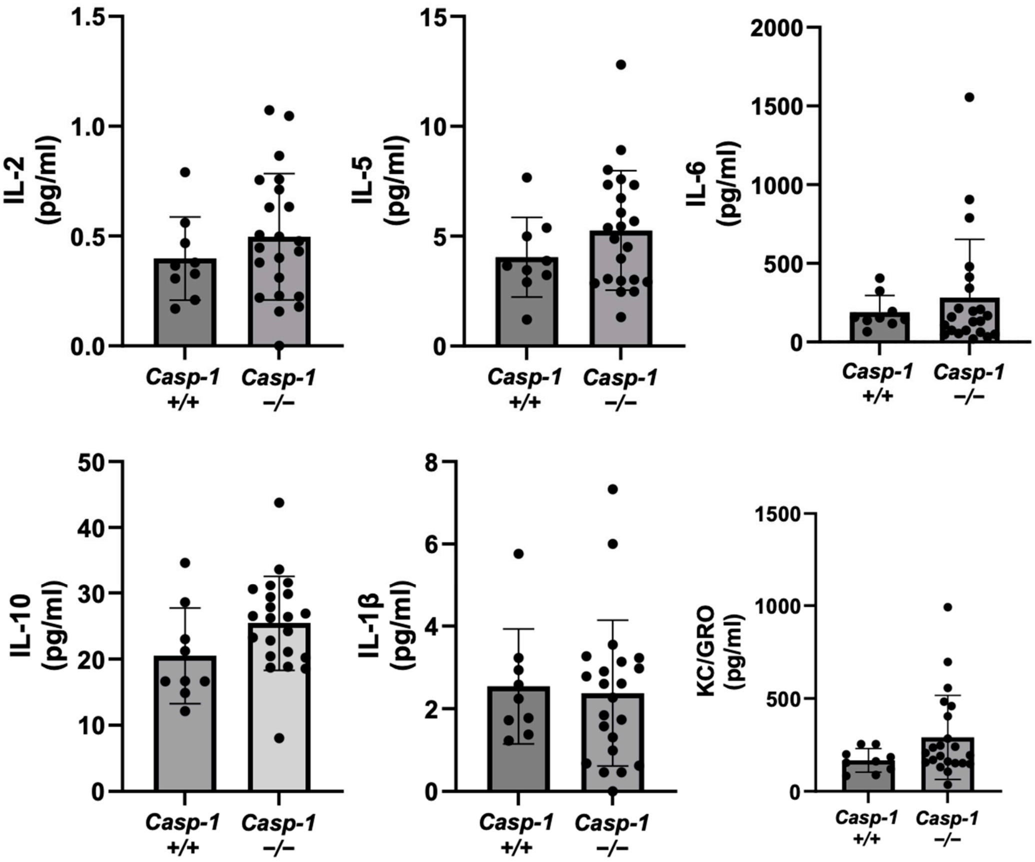

Figure S2. Proinflammatory cytokines detected serum from *Tg26$^{+/–}$/ApoE$^{–/–}$/Casp-1$^{+/+}$* and *Tg26$^{+/–}$/ApoE$^{–/–}$/Casp-1$^{-/-}$* mice on 12 weeks of an atherogenic diet. Serum from the *Tg26$^{+/–}$/ApoE$^{–/–}$/Casp-1$^{+/+}$ (Casp-1$^{+/+}$)* and *Tg26$^{+/–}$/ApoE$^{–/–}$/Casp-1$^{–/–}$ (Casp-1$^{–/–}$)* was analyzed for different proinflammatory cytokines. Data were analyzed by two-tailed t-tests.
